# View of healthcare professionals on ultra-rapid genome sequencing and its future implementation in clinical practice for critically ill children

**DOI:** 10.1038/s41431-025-01869-y

**Published:** 2025-05-23

**Authors:** Claire Caillot, Etienne Javouhey, Stéphane Hays, Evan Gouy, Pauline Monin, Gaetan Lesca, Damien Sanlaville, Nicolas Chatron

**Affiliations:** 1 Service de génétique, Hospices Civils de Lyon, Bron, France; 2 Service d’urgences et de réanimation pédiatriques, Hôpital Femme Mère Enfant, Hospices Civils de Lyon, Bron, France; 3 Service de néonatologie et de réanimation néonatale, Hôpital de la Croix-Rousse, Hospices Civils de Lyon, Lyon, France; 4 Institut Neuromyogène, Laboratoire Physiopathologie et Génétique du Neurone et du Muscle, Equipe Métabolisme énergétique et développement neuronal, Université Lyon 1, Lyon, France

**Keywords:** Genetic testing, Genetic testing, Medical genetics, Disease genetics

## Abstract

The clinical utility of ultra-rapid genome sequencing (urGS) in neonatal and paediatric intensive care situations has been demonstrated, and barriers to its implementation in clinical practice studied. A 38-item questionnaire was distributed via medical professional learned societies to identify the views of French healthcare professionals in the field prior to its implementation. Overall, 116 responses were received: 35% from healthcare professionals working in clinical genetics, 19% in laboratory genetics, and 32% in paediatric or neonatal intensive care units (NICU/PICU). Nearly all (97%) respondents agreed that healthcare professionals should receive specific training before a first test order; 94% considered urGS useful, and 97% that the results would likely modify a decision to withdraw life-sustaining treatment. A multidisciplinary approval of the urGS request was considered necessary by 87% of respondents, and multidisciplinary discussion of the result by 84%; joint disclosing of results by a clinical geneticist and NICU/PICU physician was considered ideal for 91% of respondents, and 78% were against additional findings being disclosed at the same time as the result. For 99% of respondents, psychological assistance was crucial after the result. Based on the results, we propose a workflow to facilitate implementation in a broad range of centres.

## Introduction

Patients hospitalised in neonatal and paediatric intensive care units (NICU and PICU) frequently have underlying genetic conditions [1]. Aetiological diagnosis can have a major impact on management (specific treatment, surgical decisions, organ transplants, etc.) or on decisions specific to intensive care (intubation scheduling, withholding or withdrawal of life-sustaining treatments). While genome sequencing is progressively becoming used in routine diagnosis for rare diseases, the usual turnaround time for a genome analysis result is not compatible with patient management in intensive care units.

The definition of the turnaround time for genome sequencing varies according to which step is considered to be the first, but it is generally defined as the time between sample reception at the laboratory and the validated result. Based on the results of a randomised trial published by Kingsmore et al. in 2019, rapid genome sequencing (rGS) can be defined as a turnaround time of <2 weeks, and ultra-rapid genome sequencing (urGS) <5 days [2]. In recent years, over 20 studies on rGS or urGS as first-tier tests for critically ill children have been reported [3]. The first proof-of-concept study was published in 2012 [4]. Larger-scale prospective studies in the US and Australia have confirmed that rGS enables an earlier diagnosis than the standard genetic approach [5, 6] and has also demonstrated an impact on patient management both for conclusive and inconclusive analyses [6–8]. One notable study that illustrates the potential impact is the the case report published by Goranitis et al. that describes therapeutic adaptation, involving the introduction of thiamine (vitamin B1) treatment based on a homozygous loss-of-function variant identified in *SLC19A3*, responsible for thiamine metabolism dysfunction that caused epileptic seizures, 37 h after hospital admission for a 5-week old infant [9]. Cost-effectiveness has also been demonstrated; the gain was mainly due to reduced hospitalisation time, and was negatively correlated to the turnaround time of genome results, in favour of urGS [6, 7, 10]. Studies on the views of healthcare professionals and families on these rapid or ultra-rapid analyses have found good acceptance, whether results were conclusive or not [11–14]. However, at present, only a few national or regional rGS or urGS initiatives exist or are being set up (Australia, a few states in the US, the United Kingdom, Belgium) [15–17].

To implement urGS, the entire process around genome sequencing needs to be adapted to the time constraint, with steps both upstream (communication between NICU/PICU and genetics teams, test order approval and pre-test counselling) and downstream of the laboratory (discussion and disclosure of results, incidental findings management, psychological support). Previous studies have highlighted barriers and proposed solutions to adapt the workflow to the needs of rapid analyses [18–23]. In France, at the time of the present study, no laboratory provided urGS for NICU/PICU. Implementation of urGS faces important technical and organisational challenges, while the relevance of offering these analyses from the point of view of French geneticists and NICU/PICU physicians has not yet been determined. We therefore sought to assess their views on urGS and different implementation options.

## Material and methods

### Survey development and recruitment

A 38-item questionnaire was designed for healthcare professionals involved in the care of NICU/PICU patients, either working in NICUs/PICUs, clinical genetic wards, or genetic laboratories, based on a literature review and local experience. In France, variant interpretation and reporting is performed almost exclusively by medically qualified physicians, herein referred to as laboratory geneticists; this differs from many other countries, such as the UK, where clinical scientists can also carry out these tasks. Laboratory geneticists oversee the pre-analytical, analytical, and post-analytical processes for genetic tests. They can also work as clinical geneticists, combining laboratory responsibilities with patient consultation. To maximise the probability of questionnaire completion and to ensure a correct knowledge of the topic, we provided background information on genomic sequencing in rare diseases, its place in the current diagnostic strategy, and an overview of important references as an introduction to the questionnaire.

Questions were grouped into five sections: (i) respondent’s profile, (ii) knowledge about medical genetics, (iii) urGS test initiation and consent, (iv) test results and laboratory reports, (v) test result delivery and patient management. The data were collected anonymously. Two clinical geneticists and a laboratory geneticist with experience of the topic proofread the final questionnaire. We estimated that the survey would take 10 min to complete (Supplementary File 1).

The majority of questions used Likert scale-type answers to obtain the respondents’ opinions. Proposition ranking, multiple-entry tables, and conditional branching were also used. Free-text responses were possible in each section.

The questionnaire was validated by the Lyon University Hospital Ethics Committee in accordance with the French legislation, and distributed via medical learned societies.

### Statistics and data analyses

Data were collected and stored using a LimeSurvey system [24] hosted on a server of the University Claude Bernard Lyon 1. Responses were collected from July to October 2023. Only fully completed questionnaires were analysed. Descriptive statistics were computed for all items: either with the total number of respondents or with subgroups of physicians based on the medical area of practice. Free-text replies were analysed manually and the points made were reported if they were mentioned more than once.

## Results

### The profile of respondents

Among the 116 respondents, 94% (*n* = 109) worked in a university-affiliated hospital located throughout France (24 cities), consistent with the localisation of NICU/PICU and genetics departments. A third (32%, *n* = 37) worked in either a NICU or PICU (all were either physicians or medical residents), 35% (*n* = 40) worked in the field of clinical genetics (including a psychologist and a genetic counsellor), 19% (*n* = 22) worked in a genetics laboratory, and 10% (*n* = 12) practiced in both a clinical genetics department and a genetics laboratory. In addition, 4 respondents (all physicians) self-identified as working in non-NICU/PICU paediatric wards and 1 (research assistant) worked in clinical research; these were grouped in the Other category. Among the 116 respondents, 55% (*n* = 64) were physicians with >5 years’ experience, while 29% (*n* = 34) had <5 years’ experience; 13% (*n* = 15) were medical residents, 5 of whom were in their final year of training. Thus, over half of the respondents can be considered as experienced in their respective fields. The majority of respondents (86%, *n* = 100) were involved in NICU/PICU patient care, and 83% (*n* = 96) were involved in genetic test ordering (Table 1).

**Table 1:** Profile of respondents.

	Total number of respondents *n* = 116
Area of practice, *n* (%)	
Paediatric/neonatal intensive care	37 (32)
Clinical genetics	40 (35)
Combined clinical and laboratory genetics	12 (10)
Laboratory genetics	22 (19)
Other	5 (4)
Status, *n* (%)	
Physician >5 years’ experience	64 (55)
Physician <5 years’ experience	34 (29)
Medical resident	15 (13)
Other	3 (3)
Frequency in NICU/PICU patient care involvement, *n* (%)	
Very often (>20 times/year)	46 (40)
Often (6 to 20 times/year)	26 (22)
Sometimes (<6 times/year)	28 (24)
Never	16 (14)
Frequency of genetic test ordering, *n* (%)	
Often (>1 time/month)	70 (60)
Sometimes (<1 time/month)	26 (23)
Never	20 (17)

The ‘Other’ category for area of practice is composed of non-NICU/PICU paediatric wards and a clinical research activity. The ‘Other’ category in status corresponds to a clinical research associate, a psychologist, and a genetic counsellor. Participants were asked to categorise the frequency of their involvement in patient critical care and genetic test ordering. *NICU* neonatal intensive care unit, *PICU* Paediatric intensive care unit.

The four physicians who declared that they worked in a non-NICU/PICU paediatric ward and the three non-medical healthcare professionals (research assistant, psychologist, genetic counsellor) were grouped together in the ‘Other specialty’ category. The latter was considered too heterogeneous for subgroup analysis, which therefore concerned physicians (including residents) working in NICU/PICU (*n* = 37), clinical genetics (*n* = 38), combined clinical and laboratory genetics (*n* = 12), and laboratory genetics (*n* = 22).

### Medical genetics knowledge and training

**Fig. 1:**
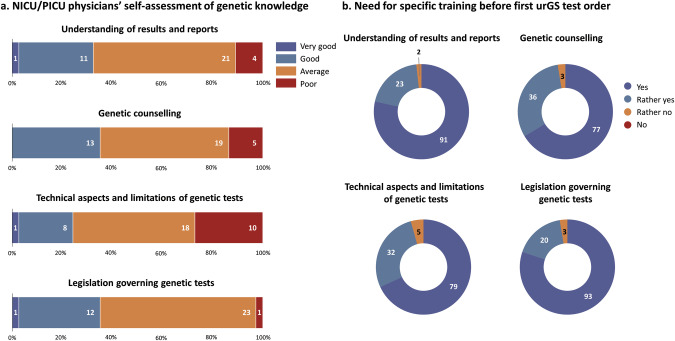
Medical genetics knowledge and training. **a** Self-assessed level of medical genetics knowledge of NICU/PICU physicians. **b** Perceived need for specific training before first urGS test ordering expressed by all participants. NICU neonatal intensive care unit, PICU paediatric intensive care unit.

To evaluate the need for specific training, respondents were asked to self-evaluate their knowledge on medical genetics, including legal aspects, technical aspects and limitations of genetic tests, understanding of genomic reports, and genetic counselling. Across the 4 aspects, a mean 97% of clinical geneticists and laboratory geneticists (both of which are physicians in France) considered their knowledge of genetics as ‘Very good’ or ‘Good’. In contrast, a mean of 55% of the NICU/PICU physicians considered their knowledge level as ‘Average’ and 14% as ‘Poor’ (Fig. 1a).

When asked if specific training was necessary before the first urGS test order for NICU or PICU patients, a mean 97% of respondents agreed that healthcare professionals should receive specific training before a first test order (Fig. 1b), this view being shared across the subgroups. Three respondents commented that the training should be basic knowledge tailored to their field of practice, in order to give them the minimal knowledge useful to them.

### Perceived utility and possible impact

**Fig. 2:**
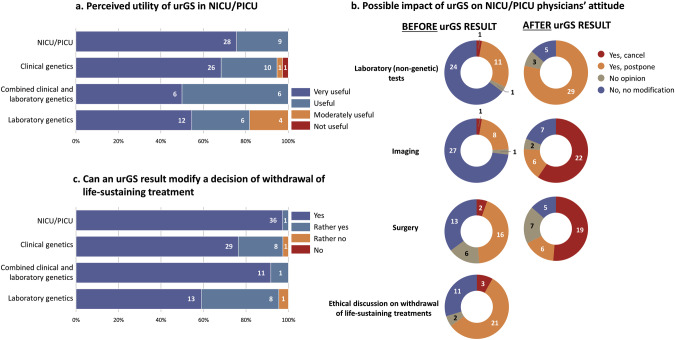
Perceived utility and potential impact of urGS. **a** Perceived utility of urGS expressed by subgroups, excluding the ‘Other speciality’ category. **b** Potential impact on laboratory (non-genetic) and imaging tests ordering, surgery, and ethical discussion for the NICU/PICU physicians subgroup only. **c** Potential impact on decision to withdraw life-sustaining treatments expressed by subgroups, excluding the ‘Other speciality’ category. urGS ultra-rapid genome sequencing, NICU neonatal intensive care unit, PICU paediatric intensive care unit.

Nearly all respondents (94%, *n* = 109/116) perceived urGS ‘Very useful’ or ‘Useful’ in a NICU/PICU situation, and subgroups data are presented in Fig. 2a. Six respondents considered urGS as ‘Moderately useful’; five were geneticists (laboratory or clinical), and one was a paediatric specialist (a dermatologist). A single respondent (a clinical geneticist who cared for NICU/PICU patients <6 times/year) reported that the urGS was not useful (Fig. 2a).

The majority of NICU/PICU physicians, who are more likely to order additional tests, said they would not cancel or postpone a laboratory (non-genetic; 65% [24/37]) or an imaging test order (73% [27/37]) while waiting for urGS results. However, 65% (24/37) would cancel or postpone an ethical discussion on the decision to withhold or withdraw life-sustaining treatments. Forty-nine percent (18/37) said they would cancel or postpone surgery, while 35% (13/37) said they would not modify the surgery. After reception of the urGS results, the majority would cancel imaging test orders (60% [22/37]) or surgery (51% [19/37]; Fig. 2b). Ninety-seven percent (112/116) of all the respondents considered that a genome result (conclusive or not) could alter the decision to withhold or withdraw life-sustaining treatments. Detailed data for subgroups are presented in Fig. 2c. Nine participants commented that it was difficult to answer, as the question was not focused on a specific situation.

### Organisation of ultra-rapid genome sequencing test order

Ninety-three percent of respondents (108/116) agreed that a dedicated workflow with designated ‘champions’ in each ward would be a relevant organisation.

Respondents were asked to select from a list which indications would lead to them ordering urGS: 82% (95/116) selected neonatal hypotonia, 78% (91/116) multiple malformation syndromes, 78% (91/116) neonatal epilepsy, 58% (67/116) prematurity with atypical complications, and 57% (66/116) for cardiac diseases. Twenty-eight percent (32/116) said they would order an urGS in the presence of additional indications, most frequently suspicion of a metabolic disorder (11/29 free-text answers). Four participants reported that it was difficult to identify a specific indication, as they believed urGS would be more useful to guide management in situations involving short-term resuscitation or surgical considerations.

**Fig. 3:**
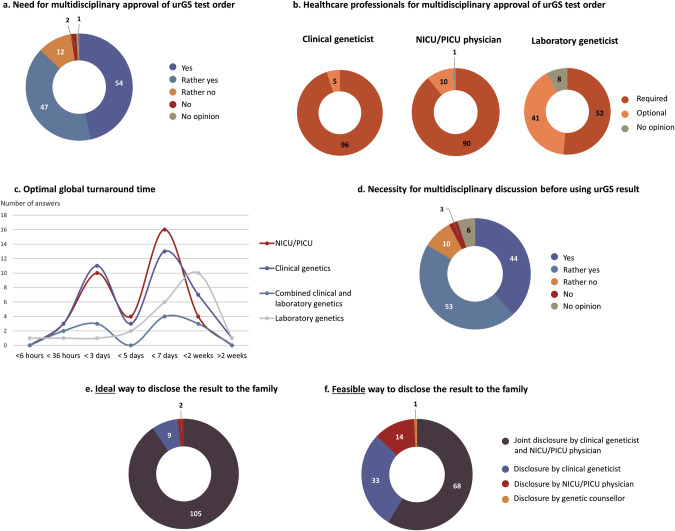
urGS organisation. **a** Need for multidisciplinary approval of urGS test orders expressed by all respondents. **b** Healthcare professionals considered as required or optional for multidisciplinary urGS test order approval by those in favour of a multidisciplinary approval. **c** Optimal turnaround time from the moment urGS has been ordered to the laboratory report, for each subgroup excluding the ‘Other speciality’ category. **d** Need for multidisciplinary discussion before using urGS result expressed by all respondents. **e** Ideal way to disclose the result to the family expressed by all respondents. **f** Feasible way to disclose the result to the family expressed by all respondents. urGS ultra-rapid genome sequencing, NICU neonatal intensive care unit, PICU paediatric intensive care unit.

Eighty-seven percent of respondents (101/116) considered that a multidisciplinary approval of genome sequencing test ordering would be necessary (Fig. 3a). Among them, 95% (96/101) thought that approval from a clinical geneticist, 89% (90/101) from a NICU/PICU physician, and 51% (52/101) from a laboratory geneticist should be mandatory in multidisciplinary approval (Fig. 3b). The majority (71%, 72/101) of respondents who considered that a multidisciplinary approval was necessary ranked a meeting (in-person or virtual) as the best option for this approval, and 62% (63/101) ranked a digital multidisciplinary meeting tool for asynchronous assessment by each professional as the second-best option. Two participants commented that email exchange was not a good option for approval. Twelve percent (14/116) were not in favour of a multidisciplinary approval of the test: five were clinical geneticists, and two laboratory geneticists.

### Consent and psychological assistance

The majority of the clinical and laboratory geneticists (90%, 65/72) reported that a clinical geneticist was the first choice for providing pre-test information and obtaining parental (or legal representative) consent for urGS, whereas 57% (21/37) of the NICU/PICU physicians did so. A genetic counsellor was the second choice for 49% (57/116) overall, and a NICU/PICU physician was the third choice for 53% (61/116) of respondents. Fifty-four percent (20/37) of the NICU/PICU physicians reported that they thought they would be able to order the test, while giving appropriate pre-test information on the analysis and consent.

The standard consent form used in France was considered appropriate for 84% (97/116) of respondents; 14% (16/116) considered it inappropriate. Eight respondents shared their concerns about incidental findings in free text.

Eighty-one percent (94/116) of respondents thought that psychological assistance was crucial before urGS test order, 59% (68/116) during urGS test order, 91% (106/116) when the result was disclosed, and all-but-one (99%, 115/116) after the result was disclosed.

### urGS results and report content

We asked respondents to choose a time frame for receiving genome sequencing results, ranging from <6 h to >2 weeks, starting from the decision to request the sequencing. Only one respondent reported the optimal turnaround time for receiving a urGS result, starting from the moment the analysis was considered, would be <6 h, 8% (9/116) reported this to be <36 h, 24% (28/116) <3 days, 35% (41/116) <7 days, and 21% (24/116) < 2 weeks. Forty-five percent (10/22) of laboratory geneticists considered the optimal turnaround time to be <2 weeks; 11% (4/37) of NICU/PICU physicians did so (Fig. 3c).

Eighty-four percent of participants (97/116) thought that a multidisciplinary discussion of the result was necessary before using it for clinical care (Fig. 3d), 82% (95/116) were in favour of the discussion involving at least a NICU/PICU physician, a clinical geneticist and a laboratory geneticist. Two respondents disagreed with this quorum of specialists, stating that a specialist for the patient’s symptoms or the disease identified through the urGS should be involved.

**Fig. 4:**
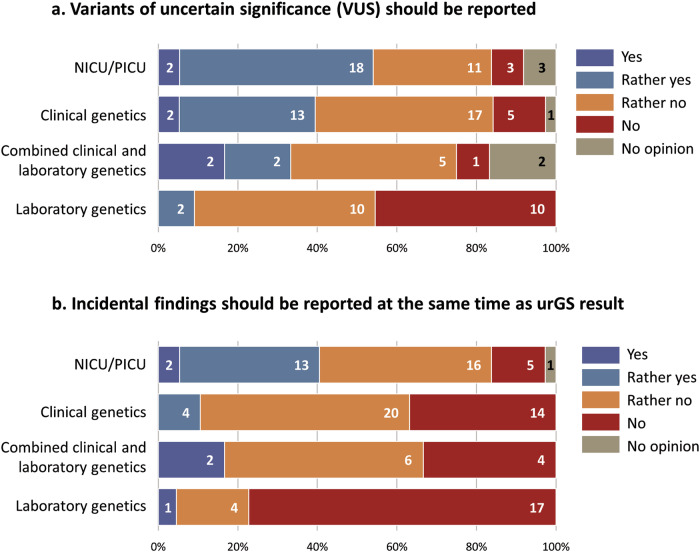
Results and report content. **a** View on reporting of VUS in the context of urGS in NICU/PICU expressed by subgroups, excluding the ‘Other speciality’ category. **b** View on reporting of incidental data at the same time as the primary urGS result expressed by subgroups, excluding the ‘Other speciality’ category. VUS variants of uncertain significance, urGS ultra-rapid genome sequencing, NICU neonatal intensive care unit, PICU paediatric intensive care unit.

Healthcare professionals were asked whether variants of uncertain significance (VUS) should be included in the report in the context of urGS in NICU/PICU: 37% were in favour (43/116), 58% (67/116) were against. Detailed data for subgroups are presented in Fig. 4a. Two respondents reported that it depended on whether the VUS was likely to become a diagnosis rapidly. Seventy-eight percent (90/116) of respondents were against incidental findings being reported at the same time as the result of the urGS in NICUs/PICU. Detailed data for subgroups are presented in Fig. 4b.

### Result disclosure to the patient and patient management

Joint disclosure by a clinical geneticist and the NICU/PICU physician was considered as the ideal way to communicate the result to the patient’s family for 91% (105/116) of respondents (Fig. 3e), but was judged to be feasible by 59% (68/116; Fig. 3f). Disclosure to the family by the clinical geneticist alone was ranked second by 73% (85/116), and by the NICU/PICU physician alone was ranked third by 58% (67/116). Disclosure by a genetic counsellor alone was ranked as the least ideal option by 74% (86/116) of respondents.

Ninety-five percent (110/116) of the respondents were in favour of informing the family prior to initiation of specific treatment following a genetic diagnosis, which could be a challenge in a NICU/PICU setting, particularly depending on who disclosed the results to the family. Finally, 89% (103/116) of respondents thought that a systematic follow-up consultation with a clinical geneticist was advisable.

## Discussion

In the present study, which aimed to collect the views of those involved regarding urGS and its implementation, respondents to the questionnaire were balanced between NICU/PICU physicians, clinical geneticists and laboratory geneticists, and respondents were from throughout France, providing a national range of opinion. However, the response rate to the survey was not possible to determine because of the way it was distributed by general learned societies. In addition, access to genetic resources remains a major problem, even beyond the proximity of a laboratory. For example, a respondent reported they had no access to clinical geneticists or genetic counsellors (*data not shown*), and we can assume that other professionals with no access to a genetics department will not have taken the time to reply to the questionnaire. It cannot also be ruled out that some respondents had prior experience of urGS while working abroad that could affect their responses, urGS was available in a very limited number of countries/centres worldwide; we therefore decided not to quantify this potential bias in order to keep the survey as short as possible, but was not mentioned in any free text comments provided by the respondents.

The first point of note from the results of the survey is that healthcare professionals believe that urGS is relevant in the context of NICUs/PICUs, and that there is a real demand from physicians for its implementation in clinical practice. Physicians seem ready to incorporate urGS for patient management, as they were likely to modify their practices while waiting for results and following the urGS results. However, the present study did not aim specifically to evaluate the impact of implementing urGS, and prospective studies of the clinical utility and cost effectiveness adapted to the French healthcare system are needed. The medical utility demonstrated in the literature [5–8, 10, 11] is, therefore, in line with the medical interest expressed via the present survey. Furthermore, the results indicate that respondents consider urGS as more relevant for NICU/PICU needs than rGS as a majority of respondents (in particular NICU/PICU physicians) consider 2 weeks (from GS test initiation to a result) to be too long in this context.

The respondents nearly all agreed on the need for training prior to the first urGS test order. The need for training in non-genetic medical specialties for the routine implementation of genetic tests has already been highlighted in the literature [25]. Although technological advances have rapidly taken place since then, training is still not optimal, as was pointed out by the NICU/PICU physicians in the present study. National training projects have emerged in countries such as Australia and England [26, 27]. In France, the French Genomic Initiative (PFMG2025) has set up a training working group to identify existing resources relevant to training in genomic medicine [28]. The training of future physicians has also improved, with the addition of genomic medicine-related courses to their initial curriculum. Although the preferred type of training (in-person or online) was not investigated herein, it would be important to discuss this locally with geneticists and to make use of existing resources for more in-depth theoretical knowledge [29, 30]. In addition, it is not easy for parents to understand the aim of urGS and what it means, especially in critical care situations [14]. However, the NICU/PICU setting can be turned into an ‘advantage’, with the continuous availability of bedside paramedical healthcare that can support parents experiencing doubts and having unanswered questions [21]; targeted training on genetic testing for these professionals appears particularly relevant.

The respondents agreed that having professional and departmental champions in each centre, as proposed in the literature, to facilitate the test initiation and organisation of rapid genetic analyses [18, 21], was important. The need for multidisciplinary approval of the test order and a discussion once the result has been obtained was expressed. This underlines the will of professionals to work closely together, and the value of having designated champions so that these discussions can take place easily and in a timely manner by relying on the existing network. A network is also mentioned in the literature as an important element for successful implementation and adoption of urGS [21].

Involvement of a clinical geneticist in pre-test information and gathering of consent was favoured as the best option by nearly all geneticists (clinical and/or laboratory), but by only just over half of NICU/PICU physicians. This is in line with the finding herein that half of NICU/PICU physicians consider that they would be able to give pre-test explanations and obtain consent. These data, together with their self-assessed mediocre knowledge of genetics, suggest that targeted training about the genetic aspects, and also the presence of a genetic counsellor, could be appropriate for the test initiation.

Having both a clinical geneticist and a NICU/PICU physician was the solution favoured by both geneticists and NICU/PICU physicians for disclosing the urGS results, although the questionnaire did not distinguish situations of conclusive or inconclusive reports. Here again, the genetic counsellor could support the NICU/PICU physician in charge of the patient in case of the unavailability of a clinical geneticist. Compared with the existing literature [31], the role of genetic counsellors could seem minor but this is probably the consequence of different legal frameworks for these professionals. In France, genetic counsellors are mainly involved in counselling families of patients with diagnosed genetic diseases and in prenatal diagnosis centres; they are rarely involved in the diagnosis of an index case. Nevertheless, their role could evolve to meet the growing needs in genetics. In particular, they could support other non-genetic physicians in providing pre-test information and consent for genetic analyses, for example, in this urGS context. This would increase the possibilities for responding quickly to requests.

Regarding the situations that could lead to urGS test order, a quarter of respondents mentioned clinical indications other than those mentioned in the questionnaire, and critical care situations rather than precise conditions were mentioned (atypical course of a simple condition, ventilatory dependence, etc.). A list of initial indications could be drawn up, but this should not be restrictive. This seems to be the approach adopted internationally, with the selection criterion being children in whom a genetic cause has been suggested [3]. However, preliminary results from an ongoing study suggest that a genetic aetiology had not been evoked initially in up to a third of neonatal intensive care patients with a genetic diagnosis [32].

The view of healthcare professionals on providing results with VUS was mixed, but the majority were against it. It is likely that adding an element of uncertainty to the patient’s already critical situation could be perceived as deleterious. In the literature, VUS of particular interest (i.e. those with a high probability of becoming a diagnosis) in the context of rGS or urGS are often discussed on a case-by-case basis in a multidisciplinary team and included in the laboratory results [3, 33]. A multidisciplinary discussion of VUS of interest could, therefore, be proposed, and decisions made on a case-by-case basis.

Genome-wide analyses may incidentally reveal variants unrelated to the initial indication, which would enable the person or the family members to benefit from preventive measures, including genetic counselling, or care. Under French law, prior to a genetic test, the patient must be informed of this possibility, and they can refuse to be informed about such findings. In the context of NICUs/PICUs, a highly stressful situation, the ability of parents to truly give free and informed consent prior to testing is probably uncertain [14, 34]. The time to report such incidental findings has also been addressed [35]. Some respondents raised the need to be cautious with incidental data, and nearly all were against providing them at the same time as the primary urGS result when the acute clinical situation, to which parental and medical attention is focused, is unrelated. We, therefore, propose that incidental data could be given at a later stage, after having the opportunity to reassess their consent on this matter.

Psychological support was considered important at all but one stage (during urGS test order) by the overwhelming majority of respondents. Parents have little time to understand the implications of urGS, and their expectations of the test are probably different from those of the medical teams. The latter aim to understand and, if possible, treat, whereas parents tend to focus on active treatment [36]. It is important that the expectations of the test are explained, and that medical, paramedical and psychological support is provided. However, there are few full-time psychologists in intensive care and genetics wards, and the present study highlights the needs expressed by NICU/PICU physicians and clinical geneticists to improve care for families in these particular situations. At the very least, we propose that a psychologist trained in genetics take part in the training of care teams, to provide them with the skills needed so that they can provide suitable support. Whenever possible, we support the involvement of either a genetics psychologist or an intensive care psychologist with specific training in the challenges of urGS. Finally, the systematic involvement of a psychologist in tandem with the geneticist/intensivist at key moments, such as when the results are disclosed or after the disclosure, should be a longer-term objective.

**Fig. 5:**
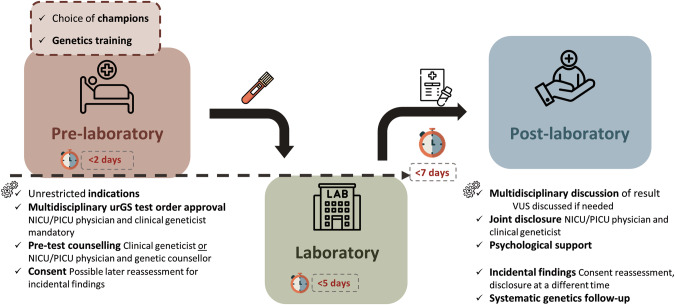
Proposed workflow for urGS in NICU/PICU setting. Prior to implementing urGS, champions for the test should be designated within each department and genetics training tailored to the field of practice should be provided. A multidisciplinary approval of urGS test orders should be used on a case-by-case basis. The notion of consent reassessment for incidental findings should be introduced. urGS results should be discussed by a multidisciplinary team for patient management. Results should be disclosed by both NICU/PICU physicians and clinical geneticists. Psychological support for parents/family should be available, especially after the results are disclosed. VUS variants of uncertain significance, urGS ultra-rapid genome sequencing, NICU neonatal intensive care unit, PICU paediatric intensive care unit.

All the points highlighted by the present study are summarised in a workflow proposal (Fig. 5). It is important to note that the present study was based on elements identified in the literature, such as the need for upfront training as well as the importance of having champions highlighted by Australian Genomics [20] and respondents had access to the main references to familiarise themselves with them. Although this may have prevented the identification of new elements, the free-text fields in the questionnaire did not reveal any. The workflow we propose is, therefore, the result of the study and the international literature.

The clinical implementation of urGS is still in its early stages. Each organisational proposal remains specific to a healthcare system and will likely require this type of study for implementation, as well as complementary studies to refine the organisation once it has been implemented. It is imperative to establish a framework while maintaining the necessary flexibility to adapt to the specific needs of NICU/PICU patients and local specificities.

The present study allowed us to define the views of French healthcare professionals on urGS and propose a workflow based on their expectations. Without overestimating the role of genetics, urGS is expected to facilitate the delivery of more personalised and effective neonatal and paediatric intensive care. Ultimately, it will open up new avenues for the diagnosis, management, and treatment of genetic conditions in newborns and children.

## Supplementary information

Questionnaire english

## Data Availability

Data available upon request.

## References

[1] French CE, Delon I, Dolling H, Sanchis-Juan A, Shamardina O, Mégy K, et al. Whole genome sequencing reveals that genetic conditions are frequent in intensively ill children. Intensive Care Med. 2019;45:627–36. 30847515 10.1007/s00134-019-05552-x PMC6483967

[2] Kingsmore SF, Cakici JA, Clark MM, et al. A randomized, controlled trial of the analytic and diagnostic performance of singleton and trio, rapid genome and exome sequencing in Ill infants. Am J Hum Genet. 2019;105:719–33. 31564432 10.1016/j.ajhg.2019.08.009 PMC6817534

[3] Kingsmore SF, Cole FS. The role of genome sequencing in the NICU. Annu Rev Genom Hum Genet. 2022;23:427–48. 10.1146/annurev-genom-120921-103442 PMC9844117 35676073

[4] Saunders CJ, Miller NA, Soden SE, Dinwiddie DL, Noll A, Alnadi NA, et al. Rapid whole-genome sequencing for genetic disease diagnosis in neonatal intensive care units. Sci Transl Med. 2012;4:154ra135. 23035047 10.1126/scitranslmed.3004041 PMC4283791

[5] Owen MJ, Niemi AK, Dimmock DP, Speziale M, Nespeca M, Chau KK, et al. Rapid sequencing-based diagnosis of thiamine metabolism dysfunction syndrome. N Engl J Med. 2021;384:2159–61. 34077649 10.1056/NEJMc2100365 PMC9844116

[6] Petrikin JE, Cakici JA, Clark MM, Willig LK, Sweeney NM, Farrow EG, et al. The NSIGHT1-randomized controlled trial: rapid whole-genome sequencing for accelerated etiologic diagnosis in critically ill infants. NPJ Genom Med. 2018;3:6. 29449963 10.1038/s41525-018-0045-8 PMC5807510

[7] Dimmock D, Caylor S, Waldman B, Benson W, Ashburner C, Carmichael JL, et al. Project baby bear: rapid precision care incorporating rWGS in 5 California children’s hospitals demonstrates improved clinical outcomes and reduced costs of care. Am J Hum Genet. 2021;108:1231–8. 34089648 10.1016/j.ajhg.2021.05.008 PMC8322922

[8] Krantz ID, Medne L, Weatherly JM, Wild KT, Biswas S, Devkota B, et al. Effect of whole-genome sequencing on the clinical management of acutely ill infants with suspected genetic disease: a randomized clinical trial. JAMA Pediatr. 2021;175:1218–26. 34570182 10.1001/jamapediatrics.2021.3496 PMC8477301

[9] Goranitis I, Wu Y, Lunke S, White SM, Tan TY, Yeung A, et al. Is faster better? An economic evaluation of rapid and ultra-rapid genomic testing in critically ill infants and children. Genet Med. 2022;24:1037–44. 35181209 10.1016/j.gim.2022.01.013

[10] Farnaes L, Hildreth A, Sweeney NM, Clark MM, Chowdhury S, Nahas S, et al. Rapid whole-genome sequencing decreases infant morbidity and cost of hospitalization. NPJ Genom Med. 2018;3:10. 29644095 10.1038/s41525-018-0049-4 PMC5884823

[11] Dimmock DP, Clark MM, Gaughran M, Cakici JA, Caylor SA, Clarke C, et al. An RCT of rapid genomic sequencing among seriously ill infants results in high clinical utility, changes in management, and low perceived harm. Am J Hum Genet. 2020;107:942–52. 33157007 10.1016/j.ajhg.2020.10.003 PMC7675004

[12] Cakici JA, Dimmock DP, Caylor SA, Gaughran M, Clarke C, Triplett C, et al. A prospective study of parental perceptions of rapid whole-genome and -exome sequencing among seriously ill infants. Am J Hum Genet. 2020;107:953–62. 33157008 10.1016/j.ajhg.2020.10.004 PMC7675003

[13] Brett GR, Martyn M, Lynch F, de Silva MG, Ayres S, Gallacher L, et al. Parental experiences of ultrarapid genomic testing for their critically unwell infants and children. Genet Med. 2020;22:1976–85. 32719395 10.1038/s41436-020-0912-4

[14] Lynch F, Nisselle A, Stark Z, Gaff CL, McClaren B. Parents’ experiences of decision making for rapid genomic sequencing in intensive care. Eur J Hum Genet. 2021;29:1804–10. 34426661 10.1038/s41431-021-00950-6 PMC8632931

[15] Stark Z, Boughtwood T, Haas M, Braithwaite J, Gaff CL, Goranitis I, et al. Australian genomics: outcomes of a 5-year national program to accelerate the integration of genomics in healthcare. Am J Hum Genet. 2023;110:419–26. 36868206 10.1016/j.ajhg.2023.01.018 PMC10027474

[16] Murch O, Jezkova J, Halstead J, Burke K, Oruganti S, Calvert J, et al. 1165 The Wales Infants’ and Children’s Genome Service’ (WINGS): diagnostic rapid whole genome sequencing for unwell children with a suspected rare genetic diagnosis. Arch Dis Child. 2021;106:A256–A256.

[17] Lumaka A, Fasquelle C, Debray FG, Alkan S, Jacquinet A, Harvengt J, et al. Rapid whole genome sequencing diagnoses and guides treatment in critically ill children in Belgium in less than 40 h. Int J Mol Sci. 2023;24:4003. 36835410 10.3390/ijms24044003 PMC9967120

[18] Stark Z, Lunke S, Brett GR, Tan NB, Stapleton R, Kumble S, et al. Meeting the challenges of implementing rapid genomic testing in acute pediatric care. Genet Med. 2018;20:1554–63. 29543227 10.1038/gim.2018.37

[19] Stark Z, Nisselle A, McClaren B, Lynch F, Best S, Long JC, et al. Attitudes of Australian health professionals towards rapid genomic testing in neonatal and paediatric intensive care. Eur J Hum Genet. 2019;27:1493–501. 31148592 10.1038/s41431-019-0429-y PMC6777457

[20] Australian Genomics Health Alliance Acute Care Flagship, Lunke S, Eggers S, Wilson M, Patel C, Barnett CP, et al. Feasibility of ultra-rapid exome sequencing in critically ill infants and children with suspected monogenic conditions in the Australian public health care system. JAMA. 2020;323:2503–11. 32573669 10.1001/jama.2020.7671 PMC7312414

[21] Best S, Brown H, Lunke S, Patel C, Pinner J, Barnett CP, et al. Learning from scaling up ultra-rapid genomic testing for critically ill children to a national level. NPJ Genom Med. 2021;6:5. 33510162 10.1038/s41525-020-00168-3 PMC7843635

[22] Franck LS, Kriz RM, Rego S, Garman K, Hobbs C, Dimmock D. Implementing rapid whole-genome sequencing in critical care: A qualitative study of facilitators and barriers to new technology adoption. J Pediatr. 2021;237:237–43.e2. 34023348 10.1016/j.jpeds.2021.05.045

[23] Wojcik MH, D’Gama AM, Agrawal PB. A model to implement genomic medicine in the neonatal intensive care unit. J Perinatol. 2023;43:248–52. 35750755 10.1038/s41372-022-01428-z PMC9789202

[24] LimeSurvey GmbH. LimeSurvey: an open source survey tool [Internet]. Hamburg, Germany. Available at: http://www.limesurvey.org

[25] Guttmacher AE, Porteous ME, McInerney JD. Educating health-care professionals about genetics and genomics. Nat Rev Genet. 2007;8:151–7. 17230201 10.1038/nrg2007

[26] Workforce education [Internet]. [cited 27 October 2023]. Available at: https://www.australiangenomics.org.au/projects/workforce-education/

[27] NHS England Genomics Education [Internet]. [cited 27 October 2023]. Available at: https://www.genomicseducation.hee.nhs.uk/

[28] Plan France Médecine Génomique 2025 - Formations [Internet]. [cited 27 October 2023]. Available at: https://pfmg2025.aviesan.fr/formations/

[29] MOOC “Diagnosing rare diseases: from the clinic to research and back” [Internet]. [cited 17 October 2023]. Available at: https://www.ejprarediseases.org/course-diagnosing-rare-diseases-from-the-clinic-to-research-and-back/

[30] BIG - Introduction à la BioInformatique et à la médecine Génomique [Internet]. [cited 17 October 2023]. Available at: https://www.fun-mooc.fr/fr/cours/big-introduction-bioinformatique-medecine-genomique/

[31] Ayres S, Gallacher L, Stark Z, Brett GR. Genetic counseling in pediatric acute care: reflections on ultra-rapid genomic diagnoses in neonates. J Genet Couns. 2019;28:273–82. 30663825 10.1002/jgc4.1086

[32] Wojcik MH, Fishler KP, Chaudhari BP. Re: “Next generation sequencing in neonatology: what does it mean for the next generation?” Hum Genet. 2023;142:161–4. 36355221 10.1007/s00439-022-02498-x

[33] Jezkova J, Shaw S, Taverner NV, Williams HJ. Rapid genome sequencing for pediatrics. Hum Mutat. 2022;43:1507–18. 36086948 10.1002/humu.24466 PMC9826377

[34] Lynch F, Prentice T, Gillam L, Stark Z, Gyngell C. Rapid genome sequencing: consent for new technologies in the neonatal intensive care context. Pediatrics. 2022;150:e2022058222. 36443237 10.1542/peds.2022-058222

[35] Robinson JO. Ask me later: deciding to have clinical exome trio sequencing for my critically ill child. Genet Med. 2021;23:1836–7. 34112998 10.1038/s41436-021-01231-9 PMC8487961

[36] Krabbenborg L, Vissers LELM, Schieving J, Kleefstra T, Kamsteeg EJ, Veltman JA, et al. Understanding the psychosocial effects of WES test results on parents of children with rare diseases. J Genet Couns. 2016;25:1207–14. 27098417 10.1007/s10897-016-9958-5 PMC5114322

