# Supplementary material for: View of healthcare professionals on ultra-rapid genome sequencing and its future implementation in clinical practice for critically ill children
Source: Eur J Hum Genet. 2025 May 23;33(7):937–44. doi: 10.1038/s41431-025-01869-y (PMC12229570; doi:10.1038/s41431-025-01869-y)
Supplement: Supplementary file 1 — Questionnaire english [file 41431_2025_1869_MOESM1_ESM.pdf]

Ultra-rapid genome sequencing in neonatal and paediatric intensive care

"Ultra-rapid" genome sequencing for rare diseases diagnosis

Genome sequencing is the most advanced genetic diagnostic test currently available. It is not currently performed as a first-tier test in France. It can take several months and up to a year to complete.

The length of time it takes is an important criterion for its relevance to neonatal and paediatric intensive care. In the literature, analysis times between receipt of the sample and issue of the first report are considered to be ultra-rapid <5 days, or rapid <2 weeks.

Currently in France, it is not generally a first-tier test, and the turnaround time is of several months within the framework of the France Médecine Génomique 2025 plan, with the SeqOIA and AURAGEN laboratories (an emergency process is currently being set up, with a expected turnaround time of 4 weeks).

The diagnostic rate varies according to the clinical situation. It is around 30 to 40% for developmental anomalies and intellectual disabilities.

What is the impact on patient management in neonatal and paediatric intensive care?

Rapid/ultra-rapid genome sequencing could help to guide management in neonatal and paediatric intensive care, notably by identifying treatable causes and clarifying short- and long-term prognosis.

Studies have been carried out abroad to assess the impact of rapid genomic analysis in this context, and have shown an impact on patient management (whether the results are positive or negative), and an improved cost-effectiveness compared to the standard approach.The perception of parents and professionals involved in patient management was also assessed, with good results.

Link to quick bibliography slideshow

Why this survey?

We would like to survey professionals involved in the care and diagnosis of patients in neonatal and paediatric intensive care units, to gather their opinions on the modalities of ultra-rapid genome sequencing (<5 days), based on points identified in the international literature. This study will enable us to identify important elements to support its implementation in clinical practice adapted to the French healthcare system.

This questionnaire contains questions on 6 themes:

- your profile,

- your knowledge of genetics,

- ultrarapid genome sequencing test order,

- consent,

- results and report,

- patient feedback and management.

This survey is part of a medical PhD, and has been validated by the HCL ethics committee. You can find the information leaflet here.

The time required to complete this survey is estimated at 10 minutes.

There are 38 questions in this survey.

Profile

"Let's begin with some introductory questions to undertsand your background!

If you have any comments on this question group, please use the comment box at the bottom of the page.

Which proposal best matches your area of practice?

*

Choose one of the following answers
Please choose **only one** of the following:

- ◯ Neonatal intensive care
- ◯ Paediatric intensive care
- ◯ Conventional paediatric ward
- ◯ Clinical genetics
- ◯ Laboratory genetics
- ◯ Combined clinical and laboratory genetics
- ◯ Other [ ]

What is your status?

*

Choose one of the following answers
If you choose 'Other:' please also specify your choice in the accompanying text field.
Please choose **only one** of the following:

- ◯ Medical resident
- ◯ Last year of medical residency
- ◯ Clinical lecturer and physician **<2 years** of practice
- ◯ Physician **<5 years** of practice
- ◯ Physician **>5 years** of practice
- ◯ Genetic counsellor
- ◯ Other ______

Which university-affiliated hospital do you practice in?

You will find the list of university hospitals below. If you do not work in any of the following hospitals, please specify the city and facility in "other"*.*

*

Choose one of the following answers
Please choose **only one** of the following:

- ◯ Amiens
- ◯ Angers
- ◯ Besançon
- ◯ Bordeaux
- ◯ Brest
- ◯ Caen
- ◯ Clermont-Ferrand
- ◯ Dijon
- ◯ Fort-de-France
- ◯ Grenoble
- ◯ La Réunion
- ◯ Lille
- ◯ Limoges
- ◯ Lyon
- ◯ Marseille
- ◯ Montpellier
- ◯ Nancy
- ◯ Nantes
- ◯ Nice
- ◯ Nîmes
- ◯ Paris
- ◯ Pointe-à-Pitre
- ◯ Poitiers
- ◯ Reims
- ◯ Rennes

- ◯ Rouen
- ◯ Saint-Etienne
- ◯ Strasbourg
- ◯ Toulouse
- ◯ Tours
- ◯ Other

Are you involved in the care of neonatal and/or paediatric intensive care patients?

*

Choose one of the following answers
Please choose **only one** of the following:

- ◯ Yes, very often (> 20 times / year)
- ◯ Yes, often (between 6 et 20 times / year)
- ◯ Yes, sometimes (<6 times / year)
- ◯ No, never

Do you have any comments on this group of questions?

Please write your answer here:

Medical genetics' knowledge

We would like to know your level of knowledge about the different aspects of genome sequencing test ordering and genetic tests in general.

If you have any comments on this question group, please use the comment box at the bottom of the page.

Do you order genetic tests in your routine clinical practice?

*

Choose one of the following answers
Please choose **only one** of the following:

- ◯ Yes, often (>1 time / month)
- ◯ Yes, sometimes (<1 time / month)
- ◯ No, never

How would you rate your knowledge of:

*

Please choose the appropriate response for each item:

	Very good	Good	Average	Poor
**The legislation governing genetic tests?**	◯	◯	◯	◯
**The technical aspects and limitations of genetic tests?**	◯	◯	◯	◯
**Understanding of results and reports?**	◯	◯	◯	◯
**Genetic counselling?**	◯	◯	◯	◯

Legally, if a pathogenic variant is identified by a genetic test, and it may affect other family members, do patients or their legal representatives have to inform them?

*

Choose one of the following answers
Please choose **only one** of the following:

- ◯ Yes
- ◯ No
- ◯ I don't know

Do you think that specific training on these subjects is necessary before the first test order by a healthcare professional of ultra-rapid genome sequencing (<5 days) in an intensive care or neonatal and paediatric situation? *

Please choose the appropriate response for each item:

	Yes	Rather yes	Rather no	No
**Legislation**	◯	◯	◯	◯
**Technical aspects and limitations**	◯	◯	◯	◯
**Understanding of results and reports**	◯	◯	◯	◯
**Genetic counseling**	◯	◯	◯	◯

Do you have any comments on this group of questions?

Please write your answer here:

Ultra-rapid genome sequencing test order

We would like to know your opinion on the relevance of ultra-rapid genome sequencing (<5 days) in neonatal and paediatric intensive care, on the organisation of the test order and on its possible impact on patient management.

If you have any comments on this question group, please use the comment box at the bottom of the page.

Do you think that ultra-rapid genome sequencing (<5 days) could be useful in a situation of neonatal/paediatric intensive care? *

Choose one of the following answers
Please choose **only one** of the following:

- ◯ Very useful
- ◯ Useful
- ◯ Moderately useful
- ◯ Not useful
- ◯ No opinion

In your opinion, what is the optimal turnaround time for receiving a genetic test result in this context, from the moment you consider that a genetic analysis is indicated? *

Choose one of the following answers
Please choose **only one** of the following:

- ◯ < 6 hours
- ◯ < 36 hours
- ◯ < 3 days
- ◯ < 5 days
- ◯ < 7 days
- ◯ < 2 weeks
- ◯ > 2 weeks

Do you think that an ultra-rapid genome sequencing test order would make you adapt your attitude in these different situations?

If you do not order clinic or paraclinic tests in your every day practice, you can select *no opinion*.

*

Please choose the appropriate response for each item:

	Yes, cancel	Yes, postpone	No, no modification	No opinion		Yes, cancel	Yes, postpone	No, no modification	No opinion
**Postpone/cancel the prescription of laboratory (non-genetic) tests?**	○	○	○	○		○	○	○	○
**Postpone/cancel imaging test order?**	○	○	○	○		○	○	○	○
**Postpone/cancel surgery?**	○	○	○	○		○	○	○	○
**Postpone/cancel an ethical discussion on withholding and withdrawal of life-sustaining treatments?**	○	○	○	○		○	○	○	○

What indications might lead you to order ultra-rapid genome sequencing (< 5 days) in neonatal/paediatric intensive care?

If you do not order clinic or paraclinic tests in your every day practice, you can select *no opinion*.

*

Please choose the appropriate response for each item:

	Yes	Rather yes	Rather no	No	No opinion
**Multiple malformation syndromes**	○	○	○	○	○
**Prematurity with atypical complications**	○	○	○	○	○
**Neonatal hypotonia**	○	○	○	○	○
**Neonatal epilepsy**	○	○	○	○	○
**Cardiac disease**	○	○	○	○	○
**Other indication (*please specify in the next question if you answered* yes *or* rather yes)**	○	○	○	○	○

Specify the indication(s):

Only answer this question if the following conditions are met:
Answer was at question ' [C04]' (What indications might lead you to order ultra-rapid genome sequencing (< 5 days) in neonatal/paediatric intensive care? If you do not order clinic or paraclinic tests in your every day practice, you can select no opinion. (
Other indication (*please specify in the next question if you answered* yes *or* rather yes)

))

Please write your answer here:

Do you consider it necessary to approve requests for ultra-rapid genome sequencing (<5 days) in the context of neonatal/paediatric intensive care on a multidisciplinary basis?

*

Choose one of the following answers
Please choose **only one** of the following:

- Yes
- Rather yes
- Rather no
- No
- No opinion

In your opinion, which healthcare professionals should be involved in the approval of ultrarapid genome sequencing test order in the context of neonatal/paediatric intensive care?

*

Only answer this question if the following conditions are met:
Answer was at question ' [C05]' (Do you consider it necessary to approve requests for ultra-rapid genome sequencing (<5 days) in the context of neonatal/paediatric intensive care on a multidisciplinary basis?)

Please choose the appropriate response for each item:

	Required	Optional	No opinion
**Inpatient department physician**	○	○	○
**Clinical geneticist**	○	○	○
**Laboratory geneticist**	○	○	○
**Intensive care paramedical team**	○	○	○
**Genetics counsellor**	○	○	○
**Psychologist**	○	○	○

What do you think would be the ideal option for this multidisciplinary approval? *

Only answer this question if the following conditions are met:
Answer was at question ' [C05]' (Do you consider it necessary to approve requests for ultra-rapid genome sequencing (<5 days) in the context of neonatal/paediatric intensive care on a multidisciplinary basis?)

All your answers must be different and you must rank in order.
Please select at most 3 answers
Please number each box in order of preference from 1 to 3

[ ] Meeting (in-person or virtual)

[ ] E-mail exchange

[ ] Multidisciplinary meeting tool for asynchronous assessment by each professional

Do you think that a dedicated workflow with dedicated champions for each profession would be appropriate for ultra-rapid sequencing (<5 days)? *

Choose one of the following answers
Please choose **only one** of the following:

- ( ) Yes
- ( ) Rather yes
- ( ) Rather no
- ( ) No
- ( ) No opinion

Do you think you could order ultra-rapid genome sequencing (<5 days) yourself, providing the necessary explanations about the test and consent to the patient or his legal representatives?

If you do not order clinic or paraclinic tests in your every day practice, you can select *no opinion*.

*

Choose one of the following answers
Please choose **only one** of the following:

- Yes
- Rather yes
- Rather no
- No
- No opinion

Do you have any comments on this group of questions?

Please write your answer here:

Consent form

Consent form, which is mandatory for genetic tests order, corresponds to a file which states that the obligation to inform relatives has been explained to the family, and records the patient's choice regarding incidental findings (pathogenic variant not linked to the pathology for which the test was ordered, for example in a gene for cancer predisposition).

This raises the question of the ability of parents of children hospitalised in intensive care units to consent in such situations, and we would like to hear your opinion on the subject.

You will find in the following link the standard consent form drafted by the French Federation of Human Genetics:

Link to FFGH standard consent

If you have any comments on this question group, please use the comment box at the bottom of the page.

In your opinion, who should give pre-test information and seek parental (or legal representative) consent for ultra-rapid genome sequencing (<5 days) in the neonatal/paediatric intensive care unit? *

All your answers must be different and you must rank in order.
Please select at most 3 answers
Please number each box in order of preference from 1 to 3

[ ] Inpatient department physician (senior or trainee)
[ ] A genetic counselor
[ ] A clinical geneticist (senior or trainee)

In your opinion, is the standard consent form (see link above) appropriate for ultra-rapid genome sequencing (< 5 days) test order?

*

Choose one of the following answers
Please choose **only one** of the following:

- ○ Yes
- ○ Rather yes
- ○ Rather no
- ○ No
- ○ No opinion

You will find the standard consent in the link in the question group description section.

Can you detail the reason(s)?

Only answer this question if the following conditions are met:
Answer was at question ' [D02]' (In your opinion, is the standard consent form (see link above) appropriate for ultra-rapid genome sequencing (< 5 days) test order?)

Please write your answer here:

Do you think the involvement of a psychologist to support the family is important: *

Please choose the appropriate response for each item:

	Yes	Rather yes	Rather no	No
**Before test initiation**	○	○	○	○
**During the test initiation period**	○	○	○	○
**After test initiation**	○	○	○	○
**When results are disclosed**	○	○	○	○
**After the results have been disclosed**	○	○	○	○

Do you have any comments on this group of questions?

Please write your answer here:

Ultra-rapid genome sequencing results and report

Genetic results are based on a 5-class classification of variants.

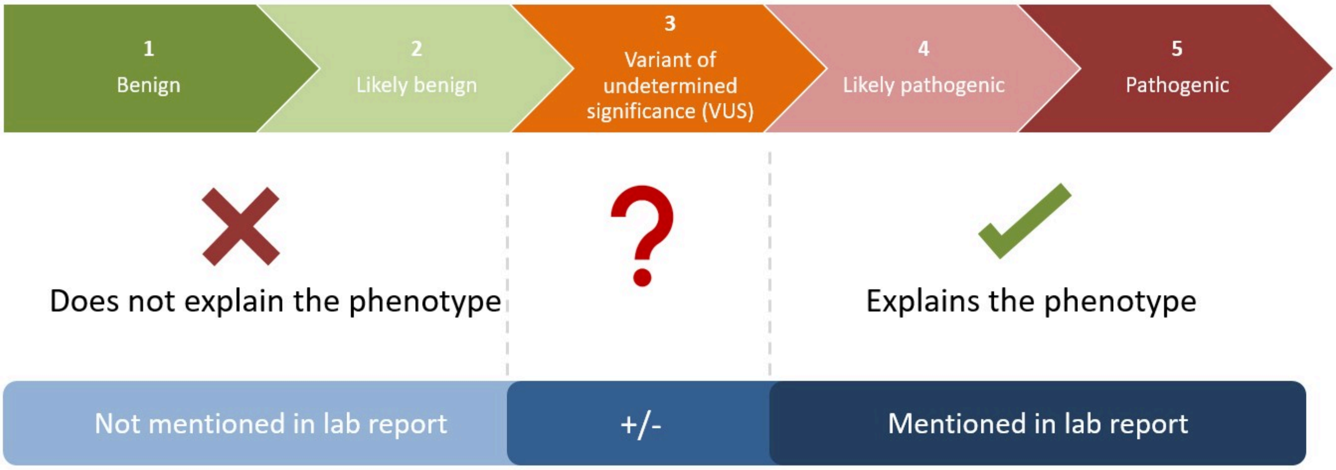

Class 3 variants are those of uncertain significance. They are not considered as responsable for the patient's pathology, and are sometimes included in the report if further studies can lead to a conclusion, in a second phase.

Furthermore, since genome sequencing enables us to look at all genes, we sometimes come across a pathogenic genetic variation that has nothing to do with the initial clinical question, for example in a gene associated with cancer predisposition. This is known as incidental findings. They are usually returned if patients have given their consent at the time of the test initiation.

In the particular context of ultra-rapid genome sequencing (<5 days) in neonatal and paediatric intensive care units, the question arises as to the relevance of reporting variants of uncertain significance (VUS) and incidental findings.

If you have any comments on this question group, please use the comment box at the bottom of the page.

Do you think variants of uncertain significance (VUS) should be reported in the context of ultrarapid genome sequencing in neonatal and paediatric intensive care? *

Choose one of the following answers
Please choose **only one** of the following:

- Yes
- Rather yes
- Rather no
- No
- No opinion

Do you think that incidental findings should be reported at the same time as the test results in this context? *

Choose one of the following answers
Please choose **only one** of the following:

- Yes
- Rather yes
- Rather no
- No
- No opinion

Do you think a multidisciplinary discussion is necessary before using the results? *

Choose one of the following answers
Please choose **only one** of the following:

- ◯ Yes
- ◯ Rather yes
- ◯ Rather no
- ◯ No
- ◯ No opinion

Do you think that a quorum of laboratory geneticists, clinical geneticists and intensive care physicians is appropriate for these results discussions? *

Only answer this question if the following conditions are met:
Answer was at question ' [E03]' (Do you think a multidisciplinary discussion is necessary before using the results?)

Choose one of the following answers
Please choose **only one** of the following:

- ◯ Yes
- ◯ Rather yes
- ◯ Rather no
- ◯ No
- ◯ No opinion

If not, which profession do you think is missing or unnecessary? *

Only answer this question if the following conditions are met:
Answer was at question ' [E03bis]' (Do you think that a quorum of laboratory geneticists, clinical geneticists and intensive care physicians is appropriate for these results discussions?)

Comment only when you choose an answer.
Please choose all that apply and provide a comment:

☐ Missing profession

☐ Profession not required

Do you have any comments on this group of questions?

Please write your answer here:

Reporting results to the patient and patient management

If you have any comments on this question group, please use the comment box at the bottom of the page.

What do you think would be the ideal way to disclose the result to the family? *

All your answers must be different and you must rank in order.

Please select at most 4 answers

Please number each box in order of preference from 1 to 4

- [ ] Joint report by clinical geneticist and inpatient department physician
- [ ] Report by the inpatient department physician
- [ ] Report by the clinical geneticist
- [ ] Report by the genetic counselor

What do you think would be a feasible way of disclosing the result to the family? *

All your answers must be different and you must rank in order.

Please select at most 4 answers

Please number each box in order of preference from 1 to 4

- [ ] Joint report by clinical geneticist and inpatient department physician
- [ ] Report by the inpatient department physician
- [ ] Report by the clinical geneticist
- [ ] Report by the genetic counselor

In your opinion, is it necessary to inform the family before starting a specific treatment following a genetic diagnosis? *

Choose one of the following answers
Please choose **only one** of the following:

- ◯ Yes
- ◯ Rather yes
- ◯ Rather no
- ◯ No
- ◯ No opinion

In your opinion, can an ultra-rapid genome sequencing test result (<5 days) modify a decision of withholding and withdrawal of life-sustaining treatments? *

Choose one of the following answers
Please choose **only one** of the following:

- ◯ Yes
- ◯ Rather yes
- ◯ Rather no
- ◯ No
- ◯ No opinion

In your opinion, is a systematic follow-up consultation with a clinical geneticist advisable? *

Choose one of the following answers
Please choose **only one** of the following:

- ◯ Yes
- ◯ Rather yes
- ◯ Rather no
- ◯ No
- ◯ No opinion

Do you have any comments on this group of questions?

Please write your answer here:

Thank you for completing this questionnaire!

If you're interested in this topic, here's the ***link to quick bibliography slideshow***

If you would like more information on this project and/or would like to receive the results of this study, please do not hesitate to contact us by e-mail:

claire.caillot@chu-lyon.fr

nicolas.chatron@chu-lyon.fr

HCL HOSPICES CIVILS DE LYON

Submit your survey.

Thank you for completing this survey.
